# Pathway to enhancing safety behavior of construction workers through subjective well-being

**DOI:** 10.3389/fpubh.2025.1652268

**Published:** 2025-09-08

**Authors:** Qi Liu, Leping Zhang, Yaxin Li

**Affiliations:** ^1^School of Marxism, Wuhan University of Science and Technology, Wuhan, China; ^2^Institute of Safety and Emergency, Wuhan University of Science and Technology, Wuhan, China

**Keywords:** subjective well-being, safety awareness, safety risk perception, work stress, safety behavior

## Abstract

**Introduction:**

This study empirically examines the influence of subjective well-being on the safety behavior of construction workers. A theoretical model is constructed based on the relationships among subjective well-being, safety awareness, safety risk perception, work stress, and safety behavior.

**Methods:**

Empirical analysis was conducted using SPSS and AMOS software on data collected from 436 valid samples to test the proposed hypotheses and model.

**Results:**

The results indicate that subjective well-being has a positive impact on the safety behavior of construction workers. Safety awareness significantly enhances the ability of workers to perceive safety risks. Both safety awareness and safety risk perception serve as significant mediators in the relationship between subjective well-being and safety behavior. Furthermore, work stress negatively moderates the relationship between subjective well-being and safety behavior.

**Discussion:**

The findings underscore the importance of promoting subjective well-being to enhance safety behavior among construction workers. The mediating roles of safety awareness and risk perception, along with the moderating effect of work stress, provide a comprehensive understanding of the underlying mechanisms. These insights offer valuable implications for developing targeted interventions to improve safety outcomes in the construction industry.

## 1 Introduction

The construction industry is characterized by a large, highly mobile workforce, harsh working environments, and significant challenges in safety management, leading to frequent fatal accidents that cause serious casualties and economic losses. According to statistics from the International Labor Organization (ILO), at least 60,000 fatal accidents occur annually in the global construction industry, equivalent to one death every 10 min (1). This sector accounts for approximately 17% of all workplace fatal accidents worldwide. In industrialized countries, while construction employs only 6–10% of the total workforce, it contributes to 25–40% of occupational deaths, highlighting the high-risk nature of the industry. Based on Heinrich’s accident causation theory, unsafe behavior of individuals and unsafe conditions of objects are the direct causes of accidents (2). However, a large number of subsequent studies (such as Reason’s Swiss Cheese Model and Hollnagel’s Functional Resonance Analysis) have shown that construction accidents are the result of systemic failures, involving interactions at multiple levels including management deficiencies, weak safety culture, organizational pressure, environmental factors, and individual behaviors (3, 4). Studies have shown that construction workers tend to prioritize identity recognition, humanistic care, and social equity (5, 6). However, they also exhibit issues such as weak safety awareness, inadequate risk perception, and increasing work stress (7), resulting in a higher incidence of unsafe behaviors and occupational injuries. Therefore, exploring the mechanisms influencing the safety behaviors of construction workers is critically important.

Research on the safety behaviors of construction workers has primarily focused on aspects such as safety awareness (8), safety climate (9), group norms (10), and working environment (11). However, there is relatively little research on the impact of well-being on the safety behaviors of construction workers. Meanwhile, globally, occupational health and safety (OHS) has expanded beyond traditional accident prevention to encompass the dimension of holistic well-being promotion (12). The International Labor Organization (ILO) defines occupational health as a “comprehensive practice aimed at promoting and maintaining the highest degree of physical, mental and social well-being of workers.” This definition transcends the narrow scope of mere physical safety, emphasizing psychological and social dimensions of well-being (13). Wu et al. (14) pointed out in their review on psychological factors in construction safety that compared with “traditional” factors such as safety climate and leadership, empirical research on the relationship between well-being and safety behavior is relatively scarce. Similarly, a systematic review by Ungaro et al. (15) also confirmed the existence of research gaps in this field and called for more exploration on how psychological states affect the safety decision-making and behaviors of workers in high-risk industries. The World Health Organization (WHO) published the “Healthy Workplaces: a model for action: for employers, workers, policy-makers and practitioners,” establishing for the first time a globally unified framework for healthy workplaces. This framework lists “promoting positive health behaviors” and “creating positive social value” as two of its six core principles, which highlights the core position of positive psychological states in the workplace and their potential association with safety performance (16). In this study, subjective well-being (SWB) is defined, in accordance with Diener’s (17–19) classic definition, as a combination of an individual’s overall and cognitive evaluation of their life quality (life satisfaction) and emotional experiences (positive emotions such as pleasure and contentment; negative emotions such as stress, anxiety, and sadness). It is not simply a synonym for “happiness” but a complex construct that includes both positive and negative dimensions. As a high-risk industry, construction workers, facing harsh working conditions and prolonged separation from their families, often place a higher pursuit of happiness than workers in other sectors. Subjective well-being is a positive emotional response or experience that significantly impacts the physical and mental health development of construction workers and has become one of the critical indicators for evaluating the construction industry comprehensively (20). Therefore, introducing the key factor of subjective well-being into the field of construction safety research is of great significance for promoting the sustainable development of the industry.

Given the relationship between subjective well-being and the safety behaviors of construction workers, simply studying the direct relationship between the two does not clarify the specific pathways and mechanisms of influence. Construction workers, as frontline personnel engaged in organizational production activities, are directly exposed to the risks of safety accidents and inevitably experience pressure from various aspects of life. A worker’s occasional emotional disturbances and dissatisfaction with life may significantly affect their safety awareness and risk perception at work, leading to unsafe behaviors. Existing studies have pointed out that work stress is an important situational factor affecting workers’ safety performance, and may moderate the relationship between psychological states and behavioral outcomes (21). However, the role of stress in the relationship between well-being and safety behavior lacks in-depth discussion in the construction field. Therefore, based on the current state of research, this study takes the subjective well-being of construction workers as the antecedent variable, safety awareness and safety risk perception as mediating variables, and introduces work stress as a moderating variable to construct a theoretical model. This study aims to investigate the pathways and mechanisms through which subjective well-being influences the safety behaviors of construction workers, thereby providing theoretical support and practical references for managers in the construction industry to improve worker safety behavior, reduce onsite accidents, and enhance safety management practices.

## 2 Theoretical framework and research hypotheses

Based on an integrative core framework combining the Affect-Driven Model and Cognitive Psychology Theory, this study investigates the intrinsic mechanism through which construction workers’ subjective well-being influences safety behavior. The Affect-Driven Model emphasizes that an individual’s emotional state directly shapes their risk assessment and motivation to respond (22). Cognitive Psychology Theory illustrates how emotional states influence risk perception and safety decision-making by affecting cognitive processes such as attention allocation, depth of information processing, and processing styles (23). These two theories provide a solid theoretical foundation for exploring the mediating pathways through which subjective well-being affects safety behavior via safety awareness and safety risk perception.

### 2.1 Direct relationship hypothesis

From the perspective of positive psychology, the role of workers’ subjective well-being in guiding behavior has received increasing attention. Diener (17) proposed in 1984 that subjective well-being reflects an individual’s satisfaction with their quality of life, based on their emotional responses and evaluative standards. Research by Diener, Suh, and Lucas (18) found that subjective well-being comprises three independent factors: positive affect, negative affect, and life satisfaction. Oishi et al. (19) argued that higher levels of subjective well-being are associated with better health and longevity, improved social relationships, work performance, and creativity. Jahan et al. (24), in their study of the influence mechanisms of subjective well-being, selected two measurement dimensions: positive and negative emotions, and life satisfaction. Considering that emotional balance can more comprehensively reflect the potential impact of an individual’s daily emotional state on safety-related cognition and behavior, and that life satisfaction, as a cognitive evaluation dimension, plays an important predictive role in work attitude and safety investment. Meanwhile, for the simplicity of the model and measurement efficiency, this study measures the subjective well-being of construction workers from two dimensions: emotional balance and life satisfaction. Among them, emotional balance refers to the relative tendency of positive and negative emotions displayed by construction workers in construction scenarios (such as working at heights and under construction period pressure). Life satisfaction focuses on the overall evaluation of construction workers on work-related aspects of life.

Hypothesis on the relationship between subjective well-being and safety behavior. Ni et al. (25), in their study targeting the new generation of construction workers, found that job satisfaction can effectively motivate safety behaviors. Santana et al. (26) explored the complex relational network between construction workers’ well-being and its related factors (such as stress and job dissatisfaction) and risk perception, emphasizing the key role of these factors in understanding intentional unsafe behaviors. Based on a sample of construction workers in Malaysia, Saleem et al. (27) confirmed that the dimensions of psychological capital (hope, self-efficacy, resilience, and optimism) have a significant positive impact on safety participation behaviors. From the perspective of self-validation theory, Yang et al. (28) found that construction workers’ age expectations (including positive expectations for physical, mental, and cognitive functions) all positively affect safety behaviors, which reflects that positive expectations for future states can promote safety behaviors. Huang et al. (29) showed that construction workers’ emotional intelligence (which affects emotional regulation ability) and emotional states significantly influence their safety performance, and pointed out that positive emotions can enhance well-being and reduce unsafe behaviors. Based on social cognitive neuroscience theory, Chong et al. (30) further found that the emotional valence of construction workers directly affects their response time to safety hazards, providing mechanistic evidence for the impact of emotional states on safety cognitive processes. The aforementioned studies indicate that in the context of the construction industry, workers’ positive psychological states (including but not limited to job satisfaction, positive emotions, positive expectations for the future, and emotional regulation ability) are significantly associated with their safety behaviors. Thus, this study proposes the following hypothesis: Subjective well-being can significantly enhance the safety behaviors of construction workers (H1).

Hypothesis on the relationship between safety awareness and safety risk perception. Safety awareness is a psychological state of vigilance that construction workers maintain in their production activities, embodying a commitment to safety in order to prevent harm to themselves or others (31). Safety risk perception refers to individuals’ subjective judgments regarding the characteristics and severity of specific risks (32). In the production process, safety risk perception encompasses the identification and evaluation of risk factors related to the workplace, personnel, equipment, and operational procedures. The impact of safety awareness on safety risk perception has been well established. Kim et al. (8) argue that construction workers need to be adept at identifying and being alert to potential danger signals that could harm themselves or others. However, if workers exhibit weak safety awareness, they may lack sufficient vigilance toward the risk factors in their surrounding environment, leading to a decline in their safety risk perception capabilities. Research by Vu et al. (33) found that during emergency evacuation processes, higher public safety awareness correlates with elevated levels of risk perception, prompting individuals to take preventive or evasive measures to mitigate personal losses. The aforementioned research indicates that safety awareness, as an intrinsic alertness-oriented cognitive state, serves as a critical antecedent for enhancing risk perception capabilities (34). Therefore, the hypothesis proposed in this study is: Safety awareness has a significant positive impact on the safety risk perception of construction workers (H2).

### 2.2 Mediating role of safety awareness and safety risk perception

Hypothesis on the relationship between subjective well-being, safety awareness, and safety risk perception. According to the affect-driven model, an individual’s emotional state can influence their assessment of and response to risk (35). Specifically, a positive emotional state enhances individuals’ approach motivation and focus on potential gains, motivating workers to engage more willingly with tasks and prioritize safety requirements (36). Conversely, a negative emotional state amplifies avoidance motivation and sensitivity to potential threats, which may lead workers to disregard safety regulations and warnings more readily, impairing effective perception of external risks (37). Furthermore, cognitive psychology theories suggest that an individual’s emotional state affects their focus on tasks and their information processing methods (38). This manifests concretely in that positive emotions broaden attentional scope (broaden-and-build theory), facilitating the detection and integrative processing of environmental risk cues (39). This enhances workers’ ability to identify hazards and implement preventive measures promptly. Negative emotions narrow attentional focus and increase cognitive load, causing attentional dispersion that impedes comprehensive and timely detection of potential risks and unsafe factors at work, thereby elevating incident probability (40). Collectively, these mechanisms elucidate how subjective well-being influences cognitive processes and safety motivation, consequently enhancing safety awareness and risk perception capabilities. Therefore, this study proposes the following hypotheses: Subjective well-being can enhance the safety awareness of construction workers (H3) and improve their safety risk perception abilities (H4).

Hypothesis on the relationship between safety awareness, safety risk perception, and safety behavior. Existing research indicates that there are direct or indirect influences between safety awareness, safety risk perception, and safety behavior. Liang et al. (38) assert that effective communication mechanisms significantly enhance workers’ safety awareness. Lee et al. (39) argue that enhancing safety awareness among construction workers can improve their focus at work, thereby promoting safer behaviors. Wong et al. (40), through their exploration of the factors influencing the intention of crane operators to engage in rule violations, demonstrated that safety awareness has a significant positive effect on the intention to commit such violations. From a risk management perspective, safety behavior can be understood as a form of risk-avoidance behavior (41). For construction workers who are often exposed to high-risk environments, their safety behaviors are typically influenced by their perceptions and understandings of the risks present in their work environment. Hannani et al. (42) used a questionnaire survey to study the impact of risk perception on workers’ unsafe behaviors, concluding that stronger risk perception capabilities correlate with lower levels of unsafe behaviors; conversely, weaker risk perception capabilities lead to higher levels of unsafe practices. Cil Muhammet et al. (43) noted that improving workers’ risk perception levels can effectively suppress unsafe behaviors. Pooladvand et al. (44), from a risk decision-making perspective, found that risk perception capabilities have a positive impact on the safety behaviors of construction workers. Based on these findings, this study proposes hypotheses H5 and H6: Safety awareness and safety risk perception significantly and positively influence the safety behaviors of construction workers.

### 2.3 Moderating role of work stress

Work stress refers to the negative responses that individuals experience in the work environment due to various physical, psychological, and social pressures (45). Existing research has found a certain degree of correlation among employees’ work stress, subjective well-being, and safety behaviors (46). The construction industry is characterized by high labor intensity, long working hours, harsh working conditions, and unstable work locations, which results in construction workers enduring high levels of work stress over extended periods. Research by Yang et al. (47) indicated that sustained high levels of work stress were significantly negatively correlated with employees’ physical health, job satisfaction, and positive emotional states. Excessive work stress can lead employees to experience more negative emotions, such as anxiety, frustration, and fatigue, while positive emotions become relatively scarce. Ehrenfreund et al. (48) suggested that negative emotional states could adversely affect an individual’s safety behavior. When construction workers experience negative emotions while at work, it may impact their work performance and attention, making them more prone to mistakes or inappropriate actions, which can trigger safety incidents. Additionally, negative emotions can influence workers’ acceptance and adherence to safety regulations, thereby increasing the risk of unsafe behaviors. Moreover, high levels of work stress may disrupt the stability of the work-life balance, reducing workers’ life satisfaction and subjective well-being, which in turn affects their motivation to engage in safety production activities. Therefore, this study proposes the following hypothesis: Work stress negatively moderates the relationship between subjective well-being and safety behavior (H7).

**Figure 1:**
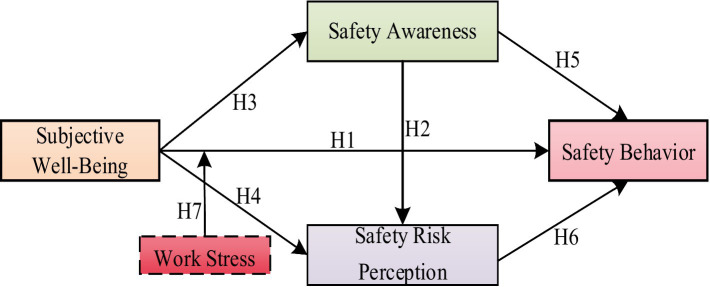
Research hypothetical model.

In summary, although existing studies have explored the relationships between subjective well-being, safety awareness, risk perception, work stress, and safety behavior, there are still the following research gaps: (1) Insufficient integration of theoretical foundations: There is a lack of studies that systematically combine the Affect-Driven Model with Cognitive Psychology Theory to explain the internal mechanisms of construction workers’ safety behavior; (2) Weak research on mediating paths: Despite preliminary evidence of links between subjective well-being and safety (25–27), the chain-mediation effect through safety awareness and risk perception (particularly their sequential interaction) remains untested in construction; (3) Insufficient exploration of boundary conditions: How the high work stress unique to the construction industry moderates the mechanism of subjective well-being on safety behavior needs further investigation. Based on the analysis above, the hypothetical model depicting the influence of subjective well-being on the safety behavior of construction workers was developed to address the existing research gaps, as shown in Figure 1.

## 3 Research design

### 3.1 Variable measurement

This study measures the variables of subjective well-being, safety awareness, safety risk perception, work stress, and safety behavior by reviewing a substantial amount of literature to select applicable and well-established scales, ensuring that the measurement instruments possess good reliability and validity. A 5-point Likert scale is employed, where 1 represents “strongly disagree” and 5 represents “strongly agree,” with values increasing accordingly.

Subjective well-being primarily consists of two measurement dimensions: emotional balance and life satisfaction. The emotional balance scale was based on the instruments developed by Kammann et al. (49) and Diener^[17]^and includes five items. The life satisfaction scale was based on the measure developed by Diener (17) and consisted of four items. The safety awareness scale was derived from Ma and He (50) scale, which contained eight items. Safety risk perception was based on the scale developed by Gyekye et al. (51), which included eight items. The work stress scale was based on the measure created by Cooper et al. (52), and consisted of eight items. The safety behavior scale was based on the instruments developed by Neal and Griffin (53) and encompassed two dimensions: safety compliance behavior and safety participation behavior, with a total of 12 items. Safety compliance behavior refers to employees’ adherence to and execution of organizational safety regulations and rules, while safety participation behavior reflects employees’ initiative and enthusiasm for engaging in safety activities and implementing safety behaviors.

### 3.2 Data collection method

To ensure the applicability and contextual relevance of the measurement scales among the Chinese construction worker population, a small-scale pilot testing was conducted with 40 construction workers at a construction site in Hubei Province prior to the formal survey. This pilot testing aimed to assess the comprehensibility of the questionnaire, the time required to complete it, and the applicability of the items. Based on the participants’ feedback (for example, confusion in understanding some terms), the research team made minor refinements to the wording of the corresponding items to make them more in line with the daily language habits and actual work scenarios of construction workers. Meanwhile, the pilot test results provided preliminary validation of the scales’ internal consistency reliability, with all Cronbach’s *α* coefficients exceeding the acceptable threshold of 0.7.

To obtain a representative sample, this study adopted stratified random sampling as the primary strategy. Specifically, 8 representative ongoing construction project sites were first selected in Hubei Province to form the sampling frame, based on geographical location and project scale. Within each selected construction site, grouping was conducted according to the available worker roster or by work type/work team. Subsequently, simple random sampling or systematic sampling methods were used to select target participants, so as to maximize the randomization of the sample. The study used paper-based questionnaires, which were administered by uniformly trained researchers who visited each selected construction site. After obtaining permission from the site management and fully explaining the purpose of the study, participants were organized to fill out the questionnaires collectively during their work breaks in designated relatively quiet areas (such as rest rooms and canteens). Researchers provided necessary on-site guidance and answered questions, and the average time to complete the questionnaire was approximately 15–20 min. The study strictly adhered to ethical guidelines: ① Written Informed Consent: Before filling out the questionnaire, each participant was given a detailed explanation of the purpose, content, voluntariness, anonymity, and confidentiality principles of the study, and a signed written informed consent form was obtained from each participant; ② Anonymity and Confidentiality: The questionnaire was designed not to collect any personally identifiable information. All collected data would only be used for academic research purposes, and strict confidentiality was guaranteed; ③ Voluntary Participation: Participants were informed that they could withdraw from the survey at any time without providing a reason.

## 4 Empirical analysis

### 4.1 Research sample

This study targeted construction workers at multiple construction sites in Hubei Province, with a total of 500 questionnaires distributed. After collection, 64 invalid questionnaires were excluded based on pre-set strict criteria. The invalid questionnaires mainly included cases such as large-scale uncompleted responses (more than 15% of the items left unanswered), obvious regular response patterns (e.g., selecting the same option for 10 or more consecutive questions), and logically contradictory responses (e.g., simultaneous selection of mutually exclusive items). Finally, 436 valid questionnaires were obtained, with an effective recovery rate of 87.2%.

Among the participants, 87.4% were male and 12.6% were female. In terms of age distribution, 2.8% were under 20 years old, 90.8% were between 21 and 45 years old, and 6.4% were over 45 years old, indicating a reasonable age structure. Regarding education levels, 37.9% had completed junior high school or lower, 27.8% had completed high school or vocational school, and 34.3% had an associate degree or higher. In terms of marital status, 29.2% were unmarried, 68.3% were married, and 2.5% were divorced. As for work experience, 14.4% had less than 3 years of experience, 59.4% had 3 to 10 years, and 26.2% had more than 10 years. The survey encompassed nine different trades, including scaffolders, rebar workers, plasterers, and electricians. The demographic characteristics of the respondents, age, educational level, and work experience, align with the basic traits of the construction worker population. Therefore, the selected participants in this study are representative of the construction workforce across various trades, ensuring the authenticity of the survey data.

### 4.2 Reliability and validity testing

Before validating the hypothesized paths in the theoretical model, it is essential to conduct reliability and validity tests on the measurement scales. The results are presented in Table 1. The factor loadings of each item ranged from 0.617 to 0.858. The Composite Reliability (CR) and Cronbach’s alpha coefficients for all measurement variables exceeded the threshold value of 0.7, and the Average Variance Extracted (AVE) for each construct was greater than 0.6. These results indicate that the scales exhibit good reliability and convergent validity. Table 2 presents the results of the discriminant validity test, showing that all the measurement factors meet the requirements for discriminant validity.

**Table 1:** Reliability and validity testing of measurement scales.

Variable	No.	Item	Factor Loading
Emotional Balance X_1_ Cronbach’s *α* = 0.823 CR = 0.857 AVE = 0.728	Y_11_	I believe my future is bright.	0.732
	Y_12_	I increasingly like myself.	0.756
	Y_13_	I always feel that the people around me are very friendly.	0.739
	Y_14_	I have always believed that difficult days will pass quickly.	0.702
	Y_15_	Compared to sadness, I am always full of smiles and laughter.	0.628
Life Satisfaction X_2_ Cronbach’s *α* = 0.838 CR = 0.923 AVE = 0.734	Y_21_	My current life is mostly close to my ideal state.	0.796
	Y_22_	I am satisfied with my work.	0.842
	Y_23_	So far, I have achieved many things that I wanted.	0.831
	Y_24_	I can balance my family and work well.	0.764
Safety Awareness Y_1_ Cronbach’s *α* = 0.702 CR = 0.811 AVE = 0.619	X_11_	I can understand all safety signs well.	0.622
	X_12_	I believe that safety issues should be prioritized at work.	0.822
	X_13_	I think it is important to conduct safety training and education.	0.737
	X_14_	I believe it is very important to consciously enhance personal safety at work.	0.809
	X_15_	I can clearly understand the hazardous factors at work.	0.710
	X_16_	I believe that accidents can be avoided.	0.688
	X_17_	I think that safety management is essential during operations.	0.858
	X_18_	I will insist on not crossing the safety red line in construction.	0.834
Safety Risk Perception Y_2_ Cronbach’s *α* = 0.744 CR = 0.828 AVE = 0.657	X_21_	I feel that my job is highly dangerous.	0.711
	X_22_	I feel that my job is very safe.	0.754
	X_23_	I feel that my job is frightening.	0.694
	X_24_	I feel that my job is risky.	0.645
	X_25_	I feel that my job is unhealthy.	0.721
	X_26_	I feel that my job may cause me harm.	0.647
	X_27_	I worry that my job will affect my physical health.	0.652
	X_28_	I feel that my job poses a risk to my life.	0.624
Work Stress Z Cronbach’s *α* = 0.850 CR = 0.926 AVE = 0.722	Z_1_	My working conditions are poor.	0.723
	Z_2_	My work is highly disruptive.	0.771
	Z_3_	My work lacks sufficient protective measures.	0.696
	Z_4_	My job content is monotonous and uninteresting.	0.825
	Z_5_	I need to work long hours and often work overtime.	0.867
	Z_6_	My work has tight deadlines and heavy tasks.	0.753
	Z_7_	My work has high demands.	0.744
	Z_8_	I am not proficient in my job operations.	0.701
Safety Compliance Behavior U_1_ Cronbach’s *α* = 0.842 CR = 0.811 AVE = 0.619	U_11_	I consistently wear necessary safety equipment at work.	0.726
	U_12_	I strictly follow safety operating procedures and regulations at work.	0.752
	U_13_	I always strive to maintain high safety standards for myself at work.	0.734
	U_14_	I do not perform operations I am not familiar with at work.	0.718
	U_15_	I do not underestimate safety operating procedures just because I am too familiar with the job.	0.745
	U_16_	I do not neglect safety operating procedures due to rushing work.	0.732
Safety Participation Behavior U_2_ Cronbach’s *α* = 0.825 CR = 0.894 AVE = 0.721	U_21_	I believe that safety operating procedures and regulations are very important.	0.748
	U_22_	I actively participate in creating a safe and civilized worksite.	0.727
	U_23_	I often attend safety education training and daily safety meetings.	0.702
	U_24_	I frequently help my colleagues address and resolve safety hazards.	0.747
	U_25_	I take the initiative to report safety hazards found on the construction site to my supervisors.	0.649
	U_26_	I provide ideas and suggestions for safe construction to my colleagues and supervisors.	0.634

It should be noted that some items (e.g., Y15, X11) had factor loadings slightly below 0.7, but were retained to preserve measurement integrity, as their removal did not significantly improve Composite Reliability (CR) or Average Variance Extracted (AVE) values.

**Table 2:** Differential validity test.

	X_1_	X_2_	Y_1_	Y_2_	Z	U_1_	U_2_
X_1_	0.826						
X_2_	0.440	0.882					
Y_1_	0.322	0.346	0.847				
Y_2_	0.402	0.426	0.452	0.854			
Z	0.245	0.264	0.284	0.366	0.812		
U_1_	0.341	0.334	0.373	0.396	0.486	0.837	
U_2_	0.243	0.294	0.238	0.259	0.243	0.319	0.861

X_1_ (Emotional Balance); X_2_ (Life Satisfaction); Y_1_ (Safety Awareness); Y_2_ (Safety Risk Perception); U_1_ (Safety Compliance Behavior); U_2_ (Safety Participation Behavior).

### 4.3 Model analysis and hypothesis testing

**Figure 2:**
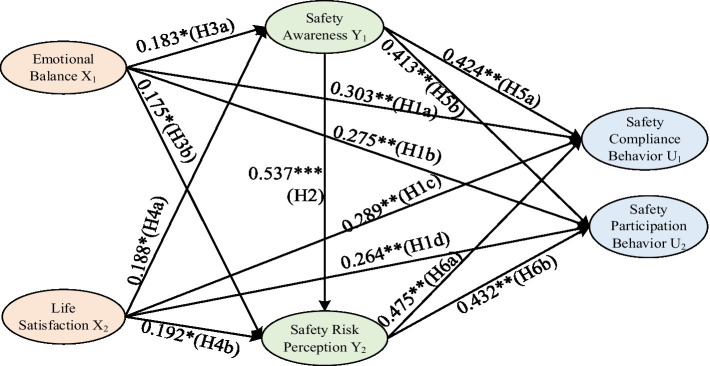
Structural equation path model.

The results of the hypothesis model path testing for this study are illustrated in Figure 2. The overall fit indices of the model meet the required criteria (χ^2^/df = 2.017, RMSEA = 0.052, GFI = 0.923, AGFI = 0.901, CFI = 0.920, TLI = 0.929, IFI = 0.926).

#### 4.3.1 Direct effect test

According to the results of the standardized analysis from the model shown in Figure 2, it can be observed that the emotional balance of construction workers has a significant positive impact on both their safety compliance behavior and safety participation behavior (β_1_ = 0.303, *p* < 0.01; β_2_ = 0.275, *p* < 0.01), confirming hypotheses H1a and H1b. Additionally, the life satisfaction of construction workers also positively influences both their safety compliance behavior and safety participation behavior (β_1_ = 0.289, *p* < 0.01; β_2_ = 0.264, *p* < 0.01), thus supporting hypotheses H1c and H1d. Overall, hypothesis H1 is validated, indicating that subjective well-being can significantly enhance the safety behaviors of construction workers. Furthermore, safety awareness positively and significantly affects the safety risk perception of construction workers (β = 0.537, *p* < 0.001), thereby supporting hypothesis H2.

#### 4.3.2 Mediating effect test

Based on the standardized analysis results from Figure 2, it is evident that the emotional balance of construction workers has a significant positive influence on both safety awareness and safety risk perception (β_1_ = 0.183, *p* < 0.05; β_2_ = 0.175, *p* < 0.05), validating hypothesis H3 (H3a and H3b). Similarly, life satisfaction positively and significantly affects both safety awareness and safety risk perception (β_1_ = 0.188, *p* < 0.05; β_2_ = 0.192, *p* < 0.05), thereby confirming hypothesis H4 (H4a and H4b). Additionally, safety awareness significantly positively influences both safety compliance behavior and safety participation behavior (β_1_ = 0.424, *p* < 0.01; β_2_ = 0.413, *p* < 0.01), confirming hypothesis H5 (H5a and H5b). Moreover, safety risk perception significantly positively impacts safety compliance behavior and safety participation behavior (β_1_ = 0.475, *p* < 0.01; β_2_ = 0.432, *p* < 0.01), supporting hypothesis H6 (H6a and H6b). From the analyses above, it can be concluded that hypotheses H3 to H6 are all validated, providing preliminary evidence that subjective well-being has a significant mediating effect between safety awareness, safety risk perception, and safety behavior.

Furthermore, this study employs the Bootstrap method to validate the mediating effects of safety awareness and safety risk perception. The results are presented in Table 3: the mediating effects of safety awareness and safety risk perception in the relationships between emotional balance, life satisfaction, and safety behavior comprise a total of 8 mediating pathways. Notably, the confidence intervals for these mediating effects do not include 0, indicating that the mediating effects of safety awareness and safety risk perception between subjective well-being and safety behavior are significant.

**Table 3:** Mediation effect test results.

Mediation model	Effect value	Boot standard error	95% Confidence interval	
			Lower limit	Upper limit
X_1_ → Y_1_ → U_1_	0.284	0.042	0.168	0.356
X_1_ → Y_1_ → U_2_	0.250	0.059	0.147	0.328
X_1_ → Y_2_ → U_1_	0.276	0.061	0.123	0.339
X_1_ → Y_2_ → U_2_	0.247	0.095	0.142	0.305
X_2_ → Y_1_ → U_1_	0.292	0.030	0.154	0.366
X_2_ → Y_1_ → U_2_	0.242	0.053	0.118	0.313
X_2_ → Y_2_ → U_1_	0.281	0.096	0.136	0.368
X_2_ → Y_2_ → U_2_	0.227	0.102	0.121	0.340

X_1_ (Emotional Balance); X_2_ (Life Satisfaction); Y_1_ (Safety Awareness); Y_2_ (Safety Risk Perception); U_1_ (Safety Compliance Behavior); U_2_ (Safety Participation Behavior).

#### 4.3.3 Moderating effect test

The moderating effect of work stress was tested using SPSS 24.0. Models 1 and 4 included the regression results with gender, age, educational level, marital status, work experience, daily working hours, and safety behavior as input variables. Models 2 and 5 primarily examined the impact of emotional balance on construction workers’ safety behavior under the moderation of work stress. Models 3 and 6 investigated the impact of life satisfaction on safety behavior under the same moderating condition. The results of the moderating effect tests are presented in Table 4: In models 2 and 5, the interaction between emotional balance and work stress significantly affects safety behavior (both safety compliance behavior and safety participation behavior) with path coefficients of (β_1_ = −0.238, *p* < 0.01; β_2_ = −0.312, *p* < 0.01). This indicates that work stress significantly negatively moderates the relationship between emotional balance and safety behavior, thus confirming hypotheses H7a and H7b. In models 3 and 6, the interaction between life satisfaction and work stress also showed significant effects on safety behavior (safety compliance behavior and safety participation behavior) with path coefficients of (β_1_ = −0.206, *p* < 0.01; β_2_ = −0.255, *p* < 0.01), indicating that work stress has a negative moderating effect on the relationship between life satisfaction and safety behavior, thus confirming hypotheses H7c and H7d.

**Table 4:** Moderating effect test.

Variables	Safety compliance behavior (U_1_)			Safety participation behavior (U_2_)		
	Model 1	Model 2	Model 3	Model 4	Model 5	Model 6
Gender	0.035	0.020	0.025	0.028	0.019	0.025
Age	0.044	0.013	0.016	0.052	0.041	0.016
Education Level	0.022	0.017	0.014	0.016	0.009	0.014
Marital Status	0.058	0.035	0.029	0.043	0.027	0.029
Work Experience	0.094*	0.0051	0.068	0.101*	0.063	0.068
Daily Working Hours	0.057	0.039	0.042	0.062	0.043	0.042
Emotional Balance (X_1_)		0.298**			0.252**	
Life Satisfaction (X_2_)			0.263**			0.247**
Work Stress (Z)		−0.457***	−0.472***		−0.506***	−0.469***
Interaction Term (X_1_ × Z)		−0.238**			−0.312**	
Interaction Term (X_2_ × Z)			−0.206**			−0.255**
Goodness of Fit (R^2^)	0.045	0.676	0.616	0.050	0.689	0.663
Model Significance (F)	2.166	95.442	94.305	2.238	97.011	96.420

***p < 0.001, **p < 0.01, *p < 0.05.

It is worth noting that in Model 1 and Model 4, the explanatory power of control variables for safety behavior is relatively weak (*R*^2^ = 0.045/0.050), which is consistent with expectations. Since variables such as gender and age are not core predictors, their main role is to exclude confounding effects. After adding subjective well-being and work stress, *R*^2^ significantly increased to 0.616–0.689, indicating that the key variables have strong explanatory power for safety behavior. The coefficients of the control variables are all insignificant (*p* > 0.05), suggesting that their impacts have been covered by the core variables and was not discussed separately.

**Figure 3:**
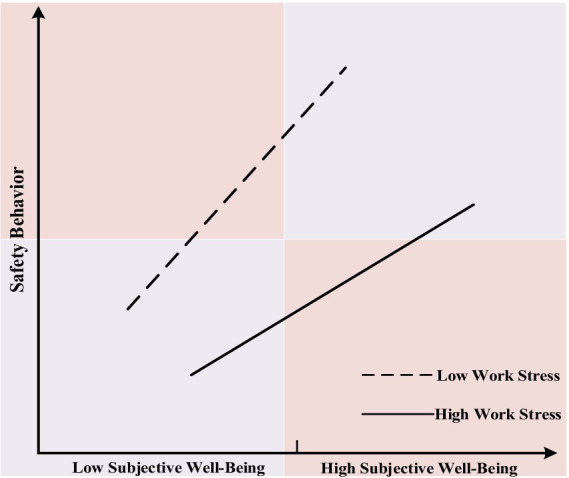
Moderating effect of work stress on the relationship between subjective well-being and safety behavior.

Consequently, the research findings suggest that work stress negatively moderates the relationship between subjective well-being and safety behavior. Using the mean values of the independent variable and the moderating variable plus and minus one standard deviation as the high-low measurement criteria, the moderating effects illustrated by the simple slope method are shown in Figure 3.

## 5 Conclusion

The subjective well-being of construction workers has a significant positive impact on their safety behaviors. The research results verify the core view of the “Affect-Driven Model” — positive emotional states enhance the motivation to respond to risks. Therefore, construction enterprises should focus on enhancing workers’ subjective well-being. First, it is essential to provide a conducive working environment. Construction companies should offer workers a safe, comfortable, and healthy work environment, which includes effective ventilation, dust and noise control measures, as well as clean, tidy, and sanitary living conditions to improve job satisfaction. Second, improving workers’ compensation is crucial. Construction enterprises can improve workers’ salary and benefits, and strengthen the behavioral driving force of subjective well-being by meeting their survival needs (54). Specifically, raising wage levels, offering reasonable overtime pay, and providing bonuses and other incentives can help workers feel that their efforts are adequately rewarded, effectively boosting their motivation and fostering a sense of belonging and loyalty to the organization. Third, granting workers adequate respect is vital. Construction companies should fully acknowledge the contributions and suggestions of workers, so as to stimulate the cognitive expansion function of emotional balance (55).

The safety awareness and safety risk perception of construction workers have a significant mediating effect between subjective well-being and safety behavior; moreover, safety awareness can effectively enhance workers’ safety risk perception. The conclusions of this study are consistent with the “triadic reciprocal determinism” framework of Social Cognitive Theory: well-being (personal factor) drives safety behavior (environmental adaptation) through cognitive evaluation (behavioral orientation) (56). Therefore, construction enterprises should focus on cultivating workers’ safety awareness and safety risk perception capabilities. First, strengthening safety education and training is essential. By regularly implementing safety education, training sessions, and drills, companies can improve workers’ safety awareness and skill levels, enabling them to properly use safety equipment and tools, become familiar with and adhere to safety operating procedures, and enhance their ability to identify and respond to potential hazards. Second, establishing a sound safety incentive mechanism is crucial. Construction enterprises can implement a reward and punishment system that recognizes and rewards workers who comply with safety regulations while penalizing and educating those who violate safety rules. This reward and punishment mechanism can motivate workers to consciously adhere to safety standards, thereby enhancing their safety awareness and sense of responsibility. Third, increasing communication and interaction among workers is important. Construction companies can organize activities that promote interaction among workers, allowing them to learn from each other’s experiences and skills. This collaborative learning environment can help raise the overall safety awareness and risk prevention capabilities of the entire team.

Work stress among construction workers negatively moderates the relationship between subjective well-being and safety behavior. The research findings are consistent with the interaction mechanism of the Job Demands-Resources Model: when work stress (a demand) is excessively high, the promoting effect of well-being (a resource) on safety behavior is weakened (57). Therefore, construction enterprises should prioritize alleviating the work-related stress experienced by their workers. First, optimizing work processes is essential. Construction companies can streamline workflows to reduce repetitive tasks and minimize wasted work time, thereby improving both efficiency and quality. This optimization can lead to a reduction in workers’ overall stress levels. Second, establishing a good communication mechanism is crucial. Construction enterprises should create effective communication channels that involve timely and meaningful interactions with workers, as well as soliciting their opinions and suggestions. This approach makes workers feel respected and valued, which can help mitigate the negative impacts of work stress. Third, attention to the mental health of workers is important. Construction companies should prioritize the psychological well-being of their employees by providing services such as psychological counseling and support. By addressing psychological issues and confusion in a timely manner, companies can enhance workers’ psychological resilience and their ability to cope with work-related stress.

Although this study provides certain theoretical and practical value for improving the well-being and safety behaviors of construction workers, it still has certain limitations. Firstly, the samples are concentrated on construction workers in Hubei Province, failing to cover the impacts of differences in economic levels, labor policies, and cultures across different regions (such as economically developed coastal areas and underdeveloped inland areas), which may to some extent limit the generalizability of the conclusions. Secondly, the theoretical model does not incorporate situational factors such as project scale and construction period urgency, especially lacking discussions on the regulatory mechanisms of these variables on the transmission path of work pressure. Finally, the research conclusions are mainly applicable to conventional construction types of work, and their applicability to high-risk special operations remains to be verified. Future research can be carried out in the following aspects: ① Conduct cross-provincial multi-construction site comparative analyses to test the regulatory role of regional economic and cultural factors; ② Introduce organizational environment variables (such as project complexity and safety investment intensity) to construct an integrated model (58); ③ Adopt diary method for tracking records (59) or longitudinal design to capture the dynamic relationship between pressure and safety behaviors.

## Data Availability

The raw data supporting the conclusions of this article will be made available by the authors, without undue reservation.
